# Female Healthcare Workers’ Knowledge, Attitude towards Breast Cancer, and Perceived Barriers towards Mammogram Screening: A Multicenter Study in North Saudi Arabia

**DOI:** 10.3390/curroncol29060344

**Published:** 2022-06-15

**Authors:** Anfal Mohammed Alenezi, Ashokkumar Thirunavukkarasu, Farooq Ahmed Wani, Hadil Alenezi, Muhannad Faleh Alanazi, Abdulaziz Saud Alruwaili, Rasha Harbi Alashjaee, Faisal Harbi Alashjaee, Abdulaziz Khalid Alrasheed, Bandar Dhaher Alshrari

**Affiliations:** 1 Department of Surgery, College of Medicine, Jouf University, Sakaka 72388, Saudi Arabia; 2 Department of Community and Family Medicine, College of Medicine, Jouf University, Sakaka 72388, Saudi Arabia; ashokkumar@ju.edu.sa; 3 Department of Pathology, College of Medicine, Jouf University, Sakaka 72388, Saudi Arabia; fawani@ju.edu.sa; 4 Department of Radiology and Nuclear Medicine, Security Forces Hospital, Riyadh 11481, Saudi Arabia; hmaalenazi@sfh.med.sa; 5 Division of Radiology, Department of Internal Medicine, College of Medicine, Jouf University, Sakaka 72388, Saudi Arabia; mfalanazi@ju.edu.sa; 6 College of Medicine, Jouf University, Sakaka 72388, Saudi Arabia; alnosairy.9@gmail.com (A.S.A.); rashaintj@gmail.com (R.H.A.); f65aisal@gmail.com (F.H.A.); drbanderalsharari@gmail.com (B.D.A.); 7 Department of General Surgery, King Saud Medical City, Riyadh 12745, Saudi Arabia; a.alrasheed@ksmc.med.sa

**Keywords:** breast cancer, risk factors, screening mammography, knowledge assessment, barriers, questionnaire examination

## Abstract

Breast cancer is the most commonly diagnosed cancer among women in the Kingdom of Saudi Arabia and other Middle East countries. This analytical cross-sectional study assessed knowledge, attitude towards breast cancer, and barriers to mammogram screening among 414 randomly selected female healthcare workers from multiple healthcare facilities in northern Saudi Arabia. Of the studied population, 48.6% had low knowledge, and 16.1% had a low attitude towards breast cancer risk factors and symptoms. The common barriers to mammogram screening were fear to discover cancer (57.2%) and apprehension regarding radiation exposure (57%). Logistic regression analysis found that lack of awareness regarding mammogram was significantly associated with age (*p* = 0.030) and healthcare workers category (ref: physicians: *p* = 0.016). In addition, we found a significant negative correlation between knowledge and barrier scores (Spearman’s rho: −0.315, *p* < 0.001). It is recommended to develop target-oriented educational programs for the healthcare workers, which would empower them to educate the community regarding the risk factors and the importance of mammogram screening. Furthermore, a prospective study is warranted in other regions of the Kingdom of Saudi Arabia to understand the region-specific training needs for the healthcare workers.

## 1. Introduction

Breast cancer is one of the significant public health issues worldwide affecting women after puberty, as stated by the World Health Organization (WHO). In 2020, there were 2.3 million cases and 685,000 deaths worldwide due to breast cancer [1]. Several international surveys reported that breast cancer causes more disability-adjusted life years among women than any other cancer in the world [2,3]. Even though the etiology for breast cancer has not been completely understood, certain risk factors, including older age, genetic predisposition, family history, early menarche, late menopause, exogenous hormone usage, and obesity, play a significant role in the breast cancer development [4,5]. In the Kingdom of Saudi Arabia (KSA), breast cancer ranks as the number one reported cancer (14.2%), and cancer-related deaths among women have an increasing annual incidence of 3.01% and a mortality of 0.93% [6,7].

Various screening methods are available for detecting breast cancer at an earlier stage, including breast self-examination, clinical breast examination, and ultrasound; however, mammography remains the primary screening modality used worldwide. In the KSA, the mammography screening technique is used for early detection of breast cancer, and it uses low-frequency X-rays to detect the features of microcalcification or mass in the breast [8,9]. As per the American Cancer Society’s guidelines, breast cancer screening to be started at 40 years of age and annual screening to be done from 45 years of age. Considering the higher incidence of breast cancer in the KSA, the Ministry of Health has recommended breast cancer screening through mammogram for all women aged from 40 to 50 years for every two years and women aged 51 to 69 years should have regular mammograms for every one to two years [10]. In the KSA, Breast Cancer Early Detection (BCED) program aims to increase early-detected cases and reduce breast cancer mortality. As per the BCED, mammogram screening is available free of cost in hospitals, selected primary health centers, and mobile clinics for Saudi nationals and public sector workers. Women are enrolled in mammogram screening programs by raising awareness through different modalities [11].

Recent epidemiological studies reported that women with inadequate knowledge regarding risk factors and susceptibility to breast cancer were less likely to accept breast cancer screening methods. Other factors associated with the low uptake of mammogram screening programs were low income, low education, and lack of information regarding available screening methods [12,13].

Healthcare workers (HCWs) play a critical role in imparting knowledge regarding available health services improving access and screening services provided by the concerned authorities. They also help in changing the behavior of an individual, families, and communities through different health-promotion activities [14,15]. Hence, it is essential to improve the knowledge and skills of the HCWs. These factors can be achieved among the HCWs by continuously assessing the current awareness, perception, and barriers towards breast cancer screening programs.

Hence, the present study assessed the knowledge and attitude towards breast cancer among the female HCWs and identified the correlation between knowledge and attitude. This study also aimed to identify the barriers to breast cancer screening programs among them.

## 2. Materials and Methods

### 2.1. Study Design and Setting

The current analytical cross-sectional survey was conducted in the Aljouf region from December 2021 to April 2022. This region is situated in the northern part of the KSA, with a total population of about 500,000. Healthcare in the Aljouf region is delivered by the ministry of health (MOH), private sectors, and other ministries through four levels: primary, secondary, specialty hospitals, and medical cities. There are 62 primary health centers, 13 general hospitals, and 2 specialty hospitals in this region under the MOH, KSA.

### 2.2. Inclusion and Exclusion Criteria

We included all the female HCW categories working in the MOH sector for a minimum duration of one year, namely doctors, nurses, pharmacists, lab technicians, and other categories. We excluded the HCWs on vacation, in different sectors (other than MOH), and those unwilling to give informed consent to participate in the present survey.

### 2.3. Sample Size Estimation

By using Cochran’s formula (n = z^2^ pq/e^2^), we estimated the minimum required female participants needed for this research. The following values were considered while calculating sample size, namely *n* = minimum size of the necessary sample, z = 1.96 at the confidence level of 95%, *p* = expected proportion (we took 50% as the *p*-value to get the maximum sample size), and *q* = 1 − *p* and e = margin of error at 5%. Considering all the above-specified values, the estimated minimum required sample size was 384. Finally, we adjusted the sample size to 480, with the expected 20% non-response rate.

### 2.4. Sampling Method

A multistage probability sampling method was performed to select the required study participants. In the first stage, we chose one general hospital, one tertiary care hospital, and all the PHCs from the Aljouf region. The research team used the lot technique to choose one general hospital and one specialty hospital from the available facilities. In the second stage, the required number of participants from each type of healthcare facility was selected based on the probability proportional to size in the following steps. Finally, we applied a systematic sampling method to select the female HCWs based on the allotted number.

### 2.5. Ethical Consideration

After obtaining ethical clearance from the Aljouf regional research ethics committee, Qurayat health affairs, MOH (approval number 126), the data collectors began the survey. The following considerations were made to avoid ethical issues:

1. Informed consent—The study participants were briefed about this study, and their willingness to participate (informed consent) was obtained.
2. Risk to the participants: There was no risk for the participants, as it was questionnaire-based research.
3. Respect for anonymity, privacy, and confidentiality: The collected details did not have any identification details of the study participants, and only the overall results of the participants were reported after the completion of the study. Hence, we maintained the anonymity of the respondents.

### 2.6. Data Collection Tool

The present study used a standardized self-administered questionnaire prepared by a team of experts from general surgery, radiology, and public health departments based on existing pieces of literature [16,17,18]. The structured questionnaire was tested for required validity and reliability. The independent experts examined the face and content validity of the data collection tool. Furthermore, we conducted a pilot study among the 30 different HCWs categories to understand the cultural adaptability and reliability in the local settings. All the pilot study participants agreed that the data collection tool was simple and easy to understand, and there were no missing data found in the completed data collection forms. The pilot study’s test score reliability coefficient (Cronbach’s alpha) for knowledge, attitude, and barrier scores were 0.78, 0.81, and 0.83, respectively, which showed good reliability in the present form of the survey questionnaire. Hence, the research team collected the data with the same data collection tool. The survey tool consisted of four sections; the first part inquired about socio-demographic details of the HCWs, including age (years), nationality, marital status, highest education qualification, current working healthcare facility, work experience duration, and HCW category. The second part consisted of sixteen questions. Of the sixteen questions, the first ten questions are related to the common risk factors (such as early puberty, late menopause, and family history), and the following six questions were related to common breast cancer symptoms. The participants were asked to choose “yes”, “no”, or “do not know”. We gave a score of one for correct answers (response as yes) and zero for wrong answers (response as no or do not know). The third part consisted of ten questions related to the attitude of the HCWs towards breast cancer and patients diagnosed with breast cancer. The participants responded on a 5-point Likert scale ranging from strongly agree to strongly disagree, and scores were given from 5 (strongly agreed) to 1 (strongly disagree). The final part included ten questions about the barriers to uptake of the mammogram screening program, including fear of the procedure, embarrassment due to breast-related tests, apprehension regarding radiation exposure, and fear of discovering cancer. The presence of a barrier was scored as one, and the absence of a barrier was scored as zero. Furthermore, the research team categorized knowledge, attitude, and barriers into high (80% and above of total scores), medium (60–79% of total scores), and low (less than 60% of total scores). Our categorization is as per the original Bloom’s cut-off point and is supported by previous studies conducted among the HCWs in the KSA and other parts of the world. In Saudi Arabia, the HCWs are expected to have high knowledge (≥80%) of common public health problems, as they play a crucial role in preventing diseases by health education and delivering healthcare services implemented by the concerned authorities [19,20]. Hence, we combined low and good scores as a single category for logistic regression analysis, and previously conducted surveys among the HCWs strongly support and justify our categorization. 

### 2.7. Data Collection Procedure

After necessary administrative approvals from the concerned healthcare facilities, the data collectors initiated the survey. We used an electronic shareable document (Google form) with the IRB-approved questionnaire for data collection. After briefing about the research rationale and objectives and obtaining informed consent, the selected HCWs were requested to fill the google form on the personal electronic device of the data collectors. For data security, the research team decided to give authorization only to the principal investigator to access, download, and export the survey’s spreadsheet. In addition, the research team made three attempts in two weeks to communicate with the selected participants. The HCWs who were unwilling to participate and those who could not be communicated with despite three attempts were recorded as non-respondents.

### 2.8. Statistical Analysis

We used the statistical package for social sciences, version 21, to export data from spreadsheets, coding, recoding, and analysis (SPSS Statistics for Windows, Armonk, NY, USA: IBM Corp.). The categorical and other descriptive results are presented as numbers (frequencies) and proportions (%), while the continuous data are presented as mean and standard deviation (SD). Initially, we performed the Shapiro–Wilk normality assumption test for the knowledge, attitude, and barriers scores. We found that all three types of scores did not meet the normality assumption (*p* < 0.001). Hence, we executed the Spearman’s correlation coefficient rank test was also used to find the strength and direction of correlation between knowledge, attitude, and barriers scores. Finally, we performed a multivariable analysis using the binomial logistic regression method to find the significantly associated socio-demographic factors with the knowledge, attitude, barriers, and awareness of the MOH, Saudi Arabia’s recommendation for mammogram screening for breast cancer. All the statistical analysis used in this research is two-tailed, and the *p*-value less than 0.05 was set as statistically significant.

## 3. Results

Of the 480 selected participants, 414 completed the survey with a response rate of 86.3%. Table 1 depicts the background characteristics of the study participants. Of the 414 responses, the majority (68.6%) are Saudi nationals, married (58.0%), bachelor’s degree holders (68.8%), nurses, and midwives (45.2%). The mean ± SD age of the studied population was 31.17 ± 6.04 years. Regarding work settings, 23.4%, 43.0%, and 33.6% of the participants worked at PHCs, general hospitals (secondary care), and tertiary care centers, respectively.

Table 2 presents the participants’ knowledge regarding breast cancer risk factors and symptoms. Regarding breast cancer risk factors, the highest proportion of correct answers was observed regarding family history of breast cancer (79.0%) and smoking (66.2%). In comparison, the lowest proportion of correct answers was seen with regards to early puberty (26.8%) and late first pregnancy (31.2%). More than three-fourths of the HCW’s correctly responded regarding change in size or shape of the breast (80.9%), non-painful lumps in the breast (75.1%), and nipple discharge (74.2%) being the risk factors of breast cancer.

We found that more than one-third of the participants had barriers in all ten categories. Barriers were commonly found as fear of discovering cancer (57.2%) and apprehension regarding radiation exposure (57%). Nearly half (48.6%) of the HCWs responded that embarrassment due to breast-related tests was their primary barrier to mammogram screening uptake (Table 3).

**Figure 1:**
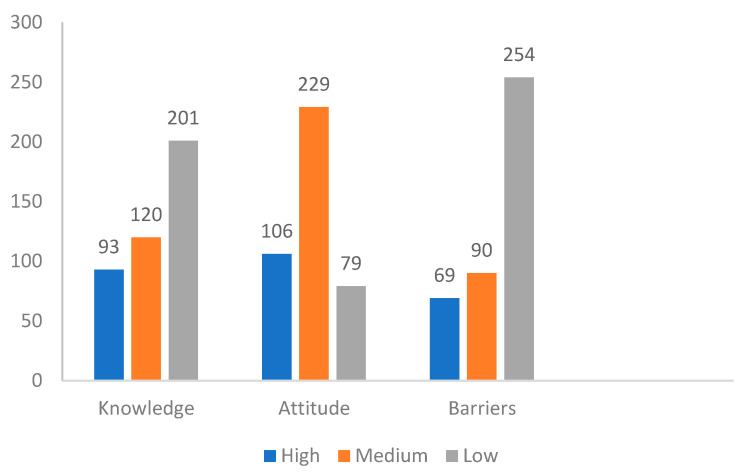
Knowledge, attitude, and barrier categories.

Of the 414 respondents, 93 (22.5%), 106 (25.6%), and 69 (16.7%) had high scores in the knowledge, attitude, and barriers categories, respectively. In comparison, 201 (79.1%), 79 (19.1%), and 254 (61.4%) had low scores in the knowledge, attitude, and barriers categories, respectively (Figure 1).

Table 4 shows the association between the knowledge subcategories and sociodemographic characteristics of the participating HCWs. Firstly, the univariate analysis was performed to compare each exposure (independent) variable with the knowledge subscales, and then, binomial logistic (multivariate analysis) were performed after adjusted with other covariables. In the univariate analysis, the characteristics that were significantly associated with the knowledge subcategories were age group (ref: up to 30 years: OR = 1.68, 95% CI = 1.06–2.67, *p* = 0.038), nationality (ref: Saudi: OR = 2.60, 95% CI = 1.62–4.19, *p* = 0.001), education (ref: diploma holders: OR = 2.96, 95% CI = 1.53–4.10, *p* = 0.001), HCWs category (ref: other categories: OR = 6.31, 95% CI = 4.91–8.10, *p* = 0.001), and family history of breast cancer (ref: no: OR = 1.93, 95% CI = 1.07–3.34, *p* = 0.037). The binomial logistic regression revealed only the following two characteristics were significantly associated with knowledge subscales, namely education status (ref: diploma holders: Adjusted OR (AOR) = 2.47, 95% CI = 1.54–4.53, *p* = 0.001) and HCW category (ref: other categories: AOR = 4.11 95%, CI = 2.86–5.76, *p* = 0.017).

Attitude subcategories and their association with sociodemographic characteristics are depicted in Table 5. The univariate analysis found that attitude subcategories were significantly associated with nationality (ref: Saudi: AOR = 1.34, 95% CI = 1.02–1.63, *p* = 0.017) and family history of breast cancer (ref: no: AOR = 2.73, 95% CI = 1.89–6.14, *p* = 0.001). However, logistic regression analysis did not reveal any significant association between independent variables and attitude subcategories.

Table 6 shows the association between barriers subcategories and sociodemographic characteristics of the participated HCWs. The binomial logistic regression revealed that only the following two characteristics were significantly associated with barriers subcategories: nationality (ref: Saudi: AOR = 1.66, 95% CI = 1.14–2.3, *p* = 0.015) and marital status (ref: married: AOR = 0.47, 95% CI = 0.28–0.69, *p* = 0.037).

Of the studied population, 66.2% were aware about the MOH, Saudi Arabia’s recommendation for mammogram screening for breast cancer In the binomial logistic regression analysis, after adjusting with other covariables of the study, we found only age (ref: up to 30 years: OR = 0.91, 95% CI = 0.83–0.97, *p* = 0.030) and HCWs categories (ref: other categories: OR = 1.83, 95% CI = 1.12–2.98, *p* = 0.001 for nurses and OR = 4.08, 95% CI = 3.01–5.79, *p* = 0.001 for physicians) were significantly associated with the awareness regarding MOH, Saudi Arabia’s recommendation for mammogram screening for breast cancer (Table 7).

The spearman’s rank correlation test revealed a significant positive correlation between knowledge and attitude scores (rho = 0.195, *p* = 0.001). In addition, we found a negative correlation between knowledge of the breast cancer risk factors and symptoms with the barriers towards uptake of mammogram screening (rho = −0.315, *p* = 0.001) (Table 8.

## 4. Discussion

In spite of the availability of these programs, a low-level mammogram screening uptake was prevalent in all sectors of the population living in all provinces of the KSA. A study conducted in the KSA reported that among 1135 women aged 50 years and above, 92% never had mammogram screening [21].

We conducted the current study among the 414 randomly selected different categories of HCWs. The present study found that a high proportion of the HCWs recognized family history and smoking as risk factors for developing breast cancer. In contrast, the lowest proportion of the participants reported early puberty, late menopause, and physical inactivity as risk factors. Similar to the current study findings, a study done by in Jordan found that female participants were highly aware of family history as a risk factor. However, only one-third recognized early puberty as a risk factor [12]. A recently conducted survey in the KSA found results in contrast to the present study. Another study done in 2020 found a lower proportion of their study participants was aware of these risk factors. The possible reason for this variation could be the difference in study settings and inclusion and exclusion criteria [16,22]. Our study revealed that nearly half of the female HCWs had low knowledge of breast cancer risk factors and symptoms regarding overall knowledge categories. A study conducted in the Riyadh region of KSA among the healthcare professionals from a tertiary care center and another study conducted in a Nigerian urban city also found similar findings [22,23].

The binomial logistic regression analysis of the current study found that knowledge scores were significantly associated with age group, nationality, level of education, and HCWs categories. Similar to the current study findings, a survey conducted among the female primary HCWs found a significant association between knowledge with the HCWs’ profession and education status [24]. The present study results revealed that only 25.2% of the HCWs had a high attitude towards breast cancer, and the attitude score was lower among the Saudi nationals than the expatriates. We could not find any other sociodemographic characteristic that was significantly associated with the attitude. Interestingly, in a Vietnam study, a higher proportion of females had a positive attitude towards breast cancer and related procedures [17]. The dissimilarities between our study and the later study are attributed to the variations in study settings, cultural characteristics, and the applied survey tools. A qualitative survey conducted in Australia reported that most of the participants had a positive perception of personalized mammogram screening, and more than 90% of them had undergone mammogram screening. The contrasting results could be due to differences in the screening model. In the KSA, the breast cancer early-detection program aims to enhance the awareness of mammogram screening through a mass approach. There is no personalized screening model available, as explored by an Australian study [25]. Even though the latter study was done in Australia, a personalized breast cancer screening model could be implemented in the KSA, where a well-established healthcare system is available as per the international standards.

Healthcare services utilization, including mammogram screening utilization by the public, is influenced by numerous barriers [16,26,27,28]. Our study revealed that more than one-third of the HCWs working in the MOH were not aware of the MOH, Saudi Arabia’s recommendation for mammogram screening for breast cancer. Moreover, the present study demonstrated that fear of discovering cancer, apprehension regarding radiation exposure, and embarrassment due to breast-related tests were the common barriers to mammogram screening uptake. Similar to the present study, some other studies conducted in the KSA and other Arab countries also reported that embarrassment due to breast examination, fear of discovering cancer, and fear of radiation were the common barriers faced by the women [16,18]. In contrast, a survey conducted in 2022 among Spanish health professionals reported that workload and financial limitations were the common barriers they faced [29]. Another study reported that being busy and lack of perceived susceptibility were the significant barriers perceived by the female HCWs [27]. The possible differences in the results might be due to the conservative nature of societies in the KSA and other Arab countries. The current study found that the barriers to mammogram screening were significantly higher among Saudi nationals, divorced/widowers, diploma holders, and other HCWs categories such as pharmacists, lab technicians, and physiotherapists. Similar to our study findings, some other authors also found that lower education is one of the predictors of barriers to mammogram screening [16,30].

Health literacy is an individual’s competence and knowledge to understand and make a proper decision on the health-related needs of them and others. A high level of health literacy and knowledge is critical for making a proper health-related decision [31,32]. The research team attempted to find the correlation between the knowledge and attitude of the HCWs to the barriers to uptake of mammogram screening. The present study results suggest that HCWs’ knowledge is negatively correlated with the barriers to uptake mammogram screening. The present study results are supported by a survey conducted in 2020 among Jordanian women [18]. In their study, more participants with a good knowledge of breast cancer reported having a mammogram than the participants with insufficient knowledge. In the KSA, the healthcare sciences curriculum is developed based on rapid change in the demography, pattern of disease, and health care needs. The healthcare students are taught about major public health problems relevant to global and local health needs. The traditional classroom learning is supplemented through additional programs such as community health activities and health promotion activities in the field. To keep up with the changing demands, the curriculum is constantly updated and incorporated into the healthcare sciences colleges’ study plan [33]. Improvements to the current training program for the HCWs and curriculum that stresses the importance of the breast cancer prevention program might facilitate them to work with greater effectiveness and improve the uptake of screening mammography by the eligible women in the community [34,35].

The WHO Global Breast Cancer Initiatives is an essential collaboration formed in 2021 to empower women and reduce breast-cancer-related deaths by 2.5% and save 25 million lives by 2040. This aim can be achieved by raising awareness of breast cancer and the importance of early detection to the HCWs, as they play a central role in strengthening the existing healthcare system [36,37].

Based on the above-mentioned findings, it is recommended to develop evidence-based and target-oriented educational programs for the HCWs, which would empower them to educate the community regarding the risk factors of breast cancer and the importance of early detection. Additionally, concerned authorities may consider changing their strategies from a mass approach to a personalized risk assessment and screening method. Furthermore, a prospective study is warranted in other regions of the KSA to understand the region-specific training needs for the HCWs.

The research team conducted the current survey using the validated tool with an adequate sample size using the standard methodology. At the same time, certain constraints need to be considered during the interpretation of this survey’s results. Firstly, this study design is cross-sectional and can find only the association, not the causation. Secondly, there is a possibility of bias due to self-reported studies present in this study. Thirdly, the present study assessed female HCWs’ knowledge, attitude, and barriers, not the general population. Hence, the current study’s findings may not be generalized to all sections of the KSA. Finally, we included only the HCWs working at the ministry of health, KSA. Nonetheless, the current research explored critical aspects of one of the significant global health issues to be addressed immediately.

## 5. Conclusions

The present study depicted that the knowledge was significantly associated with age group, nationality, level of education, and HCWs categories. However, attitude was significantly associated only with the nationality of the HCW’s. Overall, the knowledge and attitude among the northern Saudi HCWs were found to be inadequate. There were several barriers reported by the HCWs to utilizing the free mammogram services provided by the MOH, KSA, and these barriers correlated negatively with the HCW’s knowledge of breast cancer. The need of the hour is to develop evidence-based and target-oriented educational programs for the HCWs, who in turn can play a pivotal role in educating the community regarding the risk factors of breast cancer and the importance of its early detection.

## Figures and Tables

**Table 1:** Background characteristics of the female healthcare workers (HCWs) (*n* = 414).

Variables	Frequency	%
Age in years (mean ± SD)	31.17 ± 6.04	
Nationality		
Saudi	284	68.6
Non-Saudi	130	31.4
Marital status		
Married	240	58.0
Single	145	35.0
Divorced/Widowed	29	7.0
Education		
Diploma	73	17.6
Bachelors	285	68.8
Masters and above	56	15.5
Work setting		
Primary health centers (PHC)	97	23.4
General hospital	178	43.0
Tertiary care hospital	139	33.6
HCWs category		
Physicians	83	20.0
Nurse and midwifes	187	45.2
Pharmacist	43	10.5
Lab technicians	35	9.1
Other categories	66	17.2
Work experience in healthcare settings (mean ± SD)	5.91 ± 4.7	
Currently suffering from breast-related symptoms like breast pain, nipple discharge, etc.		
No	397	95.9
Yes	17	4.1
Family history of breast cancer		
No	358	86.5
Yes	56	13.5

**Table 2:** Participants knowledge regarding breast cancer risk factors and symptoms (*n* = 414).

No/Do Not Know	Yes	No/Do Not Know
	*n* (%)	*n* (%)
Risk factors		
Age—Women with age 35 years or older	249 (60.1)	165 (39.9)
First pregnancy after 30 years	129 (31.2)	285 (68.8)
Early puberty	111 (26.8)	303 (73.2)
Late menopause	151 (36.5)	263 (63.5)
Women who do not breastfeed	216 (52.2)	198 (47.8)
Obesity	202 (48.8)	212 (51.2)
Family history of breast cancer	327 (79.0)	87 (21.0)
Lack of physical activity	170 (41.1)	244 (58.9)
Smoking	274 (66.2)	140 (33.8)
Hormone therapy	271 (65.5)	143 (34.5)
Symptoms		
Non-painful lumps in the breast	311 (75.1)	103 (24.9)
Breast redness or change in color	283 (68.4)	131 (31.6)
Nipple discharge	307 (74.2)	107 (25.8)
Severe weight loss	213 (51.4)	201 (48.6)
Axillary lymph node enlargement	323 (78.0)	91 (22.0)
Change in size or shape of the breast	335 (80.9)	79 (19.1)

**Table 3:** Barriers towards uptake of mammogram screening among the participants (*n* = 414).

Barriers	Yes *n* (%)	No *n* (%)
Screening for breast cancer is not worthwhile	142 (34.3)	272 (65.7)
Apprehension regarding radiation exposure	236 (57.0)	178 (43.0)
Fear of pain related to clinical examination	231 (55.8)	183 (44.2)
Mammogram is not important	133 (32.1)	282 (67.9)
Embarrassment due to breast-related tests	201 (48.6)	213 (51.4)
Fear to discover cancer	237 (57.2)	177 (42.8)
Cancer has no cure	136 (32.9)	278 (67.1)
The test may be rejected by the family	148 (35.7)	266 (64.3)
No family history of breast cancer	154 (37.2)	260 (62.8)
Fear of not knowing the procedure	203 (49.0)	211 (51.0)

**Table 4:** Binomial regression analysis between participants’ socio-demographic characteristics and knowledge towards breast cancer (*n* = 414).

Characteristics	Total HCWs (*n* = 414)	Knowledge		Univariate Analysis Low/Medium vs. High		Multivariate Analysis * Low/Medium vs. High	
		Low/Medium (*n* = 321) *n* (%)	High (*n* = 93) *n* (%)	Unadjusted or (95% CI)	*p*-Value **	Adjusted or (95% CI)	*p*-Value **
Age (years)							
Up to 30	237	193 (81.4)	44 (18.6)	Ref		Ref	
Above 30	177	128 (72.3)	49 (27.7)	1.68 (1.06–2.67)	0.038	0.82 (0.37–1.82)	0.622
Nationality							
Saudi	284	236 (83.1)	48 (16.9)	Ref		Ref	
Non-Saudi	130	85 (65.3)	45 (34.6)	2.60 (1.62–4.19)	0.001	1.61 (0.83–3.11)	0.159
Marital status							
Married	240	179 (74.6)	61 (25.4)	Ref			
Single	145	118 (81.4)	27 (18.6)	0.67 (0.40–1.13)	0.181	0.75 (0.38–1.49)	0.408
Divorced/Widowed	29	24 (82.8)	5 (17.2)	1.01 (0.85–1.34)	0.071	0.86 (0.24–3.01)	0.809
Education							
Diploma	73	68 (93.2)	5 (6.8)	Ref		Ref	
Bachelors	285	234 (82.1)	51 (17.9)	1.85 (1.12–2.91)	0.028	2.48 (0.844–4.22)	0.099
Masters and above	56	19 (33.9)	37 (66.1)	2.96 (1.53–4.12)	0.001	2.47 (1.54–4.53)	0.001
Work setting							
PHC	97	68 (70.1)	29 (29.9)	Ref		Ref	
General hospital	178	146 (82.0)	32 (18.0)	1.34 (0.85–1.67)	0.384	0.56 (0.37–1.16)	0.117
Tertiary care hospital	139	107 (77.0)	32 (23.0)	0.87 (0.63–1.82)	0.165	0.59 (0.27–1.26)	0.173
HCWs category							
Other categories	144	130 (90.3)	14 (9.7)	Ref		Ref	
Nurse and midwifes	187	161 (86.1)	26 (13.9)	1.51 (0.75–2.99)	0.251	1.45 (0.68–3.01)	0.334
Physicians	83	30 (36.1)	53 (63.9)	6.31 (4.91–8.10)	0.001	4.11 (2.86–5.76)	0.017
Work experience in healthcare setting	5.91 ± 4.7			1.054 (1.01–1.10)	0.022	0.92 (0.72–1.66)	0.083
Family history of breast cancer							
No	358	284 (78.2)	74 (21.8)	Ref		Ref	
Yes	56	37 (73.2)	19 (26.8)	1.93 (1.07–3.34)	0.037	1.17 (0.87–2.24)	0.071

* Variables adjusted for logistic regression (enter method): Age category, nationality, marital status, education, work setting, HCWs category, work experience, and family history of breast cancer. ** Significant value less than 0.05 (two-tailed test).

**Table 5:** Binomial regression analysis between participants’ socio-demographic characteristics and attitude towards breast cancer (*n* = 414).

Characteristics	Total HCWs (*n* = 414)	Attitude		Univariate Analysis Low/Medium vs. High		Multivariate Analysis * Low/Medium vs. High	
		Low/Medium (*n* = 308) *n* (%)	High (*n* = 106) *n* (%)	Unadjusted or (95% CI)	*p*-Value **	Adjusted or (95% CI)	*p*-Value **
Age (years)							
Up to 30	237	181 (76.4)	56 (23.6)	Ref		Ref	
Above 30	177	127 (71.8)	50 (28.2)	1.29 (0.82–1.98)	0.294	1.29 (0.66–2.53)	0.452
Nationality							
Saudi	284	215 (75.7)	69 (24.3)	Ref		Ref	
Non-Saudi	130	93 (71.5)	37 (28.5)	1.34 (1.02–1.63)	0.037	1.31 (0.75–2.28)	0.348
Marital status							
Married	240	179 (74.6)	61 (25.4)	Ref		Ref	
Single	145	110 (110)	35 (24.1)	0.93 (0.68–1.51)	0.783	0.96 (0.54–1.72)	0.469
Divorced/Widowed	29	19 (65.5)	10 (34.5)	1.54 (0.58–2.68)	0.188	1.57 (0.65–3.57)	0.340
Education							
Diploma	73	56 (76.7)	17 (23.3)	Ref		Ref	
Bachelors	285	215 (75.4)	70 (24.6)	1.07 (0.79–1.67)	0.092	1.03 (0.52–2.06)	0.359
Masters and above	56	37 (66.1)	19 (33.9)	1.69 (0.78–3.68)	0.187	1.21 (3.23)	0.699
Work setting							
PHC	97	70 (72.2)	27 (27.8)	Ref		Ref	
General hospital	178	125 (70.2)	53 (29.8)	1.10 (0.64–1.90)	0.531	1.94 (0.67–2.13)	0.547
Tertiary care hospital	139	113 (81.3)	26 (18.7)	0.59 (0.42–1.43)	0.103	0.58 (0.38–1.12)	0.104
HCWs category							
Other categories	144	106 (73.6)	38 (26.4)	Ref		Ref	
Nurse and midwifes	187	148 (79.1)	39 (20.9)	0.74 (0.44–1.23)	0.245	0.71 (0.41–1.24)	0.227
Physicians	83	54 (65.1)	29 (34.9)	1.49 (0.84–2.69)	0.184	1.36 (0.65–2.86)	0.421
Work experience in healthcare setting	5.91 ± 4.7			1.01 (0.96–1.12)	0.967	0.96 (0.86–1.03)	0.092
Family history of breast cancer							
No	358	281 (78.5)	77 (21.5)	Ref		Ref	
Yes	56	27 (48.2)	29 (51.8)	3.73 (1.89–6.14)	0.001	1.28 (0.42–2.88)	0.661

* Variables adjusted for logistic regression (enter method): Age category, nationality, marital status, education, work setting, HCWs category, work experience, and family history of breast cancer. ** Significant value less than 0.05 (two-tailed test).

**Table 6:** Binomial regression analysis between participants’ socio-demographic characteristics and barriers to uptake mammogram screening (*n* = 414).

Characteristics	Total HCWs (*n* = 414)	Barriers		Univariate Analysis Low/Medium vs. High		Multivariate Analysis * Low/Medium vs. High	
		High (*n* = 160) *n* (%)	Low/Medium (*n* = 254) *n* (%)	Unadjusted or (95% CI)	*p*-Value **	Adjusted or (95% CI)	*p*-Value **
Age (years)							
Up to 30	237	94 (39.7)	143 (60.3)	Ref		Ref	
Above 30	177	66 (37.3)	111 (62.7)	1.12 (0.74–1.65)	0.624	0.79 (0.53–1.39)	0.969
Nationality							
Saudi	284	121 (42.6)	163 (57.4)	Ref		Ref	
Non-Saudi	130	39 (30.0)	91 (70.0)	2.73 (2.11–3.68)	0.005	1.66 (1.14–2.32)	0.015
Marital status							
Married	240	79 (32.9)	161 (67.1)	Ref		Ref	
Single	145	66 (45.5)	79 (54.5)	0.59 (0.39–0.76)	0.014	0.47 (0.28–0.69)	0.037
Divorced/Widowed	29	15 (51.7)	14 (48.3)	0.46 (0.31–0.59)	0.049	0.48 (0.21–1.08)	0.076
Education							
Diploma	73	33 (45.2)	40 (54.8)	Ref		Ref	
Bachelors	285	114 (40.0)	171 (60.0)	1.24 (0.74–2.01)	0.420	1.01 (0.56–1.85)	0.969
Masters and above	56	13 (23.2)	43 (66.8)	2.73 (1.27–4.91)	0.011	1.55 (0.59–3.05)	0.369
Work setting							
PHC	97	39 (40.2)	58 (59.8)	Ref		Ref	
General hospital	178	71 (39.9)	107 (60.1)	(0.61–1.68)	0.959	1.07 (0.63–1.83)	0.808
Tertiary care hospital	139	50 (36.0)	89 (64.0)	1.20 (0.70–2.04)	0.509	1.16 (0.64–2.09)	0.625
HCWs category							
Other categories	144	64 (44.4)	80 (55.6)	Ref		Ref	
Nurse and midwifes	187	75 (40.1)	112 (59.9)	1.20 (0.77–1.86)	0.428	0.58 (0.28–1.19)	0.139
Physicians	83	21 (25.3)	62 (74.7)	2.36 (1.30–4.28)	0.015	0.58 (0.28–1.21)	0.148
Work experience in healthcare setting	5.91 ± 4.7			0.99 (0.95–1.03)	0.675	0.94 (0.89–1.01)	0.068
Family history of breast cancer							
No	358	139 (38.8)	219 (61.2)	Ref		Ref	
Yes	56	21 (37.5)	35 (62.5)	1.06 (0.59–1.89)	0.850	1.04 (0.57–1.92)	0.898

* Variables adjusted for logistic regression (enter method): Age category, nationality, marital status, education, work setting, HCWs category, work experience, and family history of breast cancer. ** Significant value less than 0.05 (two-tailed test).

**Table 7:** Binomial regression analysis between participants’ socio-demographic characteristics and awareness on the MOH, Saudi Arabia’s recommendation for mammogram screening for breast cancer (*n* = 414).

Characteristics	Total HCWs (*n* = 414)	Awareness Status		Univariate Analysis No vs. Yes		Multivariate Analysis * No vs. Yes	
		No (*n* = 140) *n* (%)	Yes (*n* = 274) *n* (%)	Unadjusted or (95% CI)	*p*-Value **	Adjusted or (95% CI)	*p*-Value **
Age (years)							
Up to 30	237	93 (39.2)	144 (60.8)	Ref		Ref	
Above 30	177	47 (26.6)	130 (73.4)	1.99 (1.32– 3.01)	0.001	0.81 (0.66–0.94)	0.039
Nationality							
Saudi	284	111 (39.1)	173 (60.9)	Ref		Ref	
Non-Saudi	130	29 (22.3)	101 (77.7)	1.26 (0.82–1.95)	0.292	0.85 (0.51–1.44)	0.553
Marital status							
Married	240	66 (27.5)	174 (72.5)	Ref		Ref	
Single	145	60 (41.4)	85 (58.6)	0.72 (0.47–1.12)	0.148	1.13 (0.66–1.84)	0.665
Divorced/Widowed	29	14 (48.3)	15 (51.7)	0.87 (0.75–0.98)	0.010	0.75 (0.52–1.46)	0.061
Education							
Diploma	73	23 (31.5)	50 (68.5)	Ref		Ref	
Bachelors	285	110 (38.6)	175 (61.4)	0.79 (0.46–1.37)	0.404	0.95 (0.61–1.69)	0.133
Masters and above	56	7 (12.5)	49 (87.5)	1.81 (0.89–3.68)	0.102	1.30 (0.71–2.77)	0.076
Work setting							
PHC	97	25 (25.8)	72 (74.2)	Ref		Ref	
General hospital	178	62 (34.8)	116 (65.2)	0.81 (0.49–1.35)	0.418	1.03 (0.60–1.78)	0.521
Tertiary care hospital	139	53 (38.1)	86 (61.9)	0.67 (0.39–1.16)	0.153	0.88 (0.48–1.61)	0.682
HCWs category							
Other categories	144	73 (50.7)	71 (49.3)	Ref		Ref	
Nurse and midwifes	187	56 (29.9)	131 (70.1)	1.19 (0.74–1.91)	0.479	1.01 (0.62–1.69)	0.972
Physicians	83	11 (13.3)	72 (86.7)	2.38 (1.33–4.10)	0.003	2.12 (1.35–3.18)	0.017
Work experience in healthcare setting	5.91 ± 4.7			1.06 (1.02–1.12)	0.005	1.02 (0.96–1.09)	0.487
Family history of breast cancer							
No	358	120 (33.5)	238 (66.5)	Ref		Ref	
Yes	56	20 (35.7)	36 (64.3)	1.12 (0.72–2.91)		1.46 (0.79–2.68)	0.223

* Variables adjusted for logistic regression (enter method): Age category, nationality, marital status, education, work setting, HCWs category, work experience, and family history of breast cancer. ** Significant value less than 0.05 (two-tailed test).

**Table 8:** Correlation between knowledge, attitude, and barriers scores (Spearman’s rank correlation).

Variable	Rho */*p*-Value **
Knowledge–Attitude	0.195/0.001
Knowledge–Barrier	−0.315/0.001
Attitude–Barrier	0.060/0.226

* Spearman’s rank correlation coefficient, ** significant at 0.05 level (two-tailed).

## Data Availability

The data presented in this study are available on request from the corresponding author.
